# Extramedullary relapse of Immunoglobulin A-kappa myeloma manifesting as plasmacytoma of the pleura without bone marrow involvement and following autologous bone marrow transplant: a case report

**DOI:** 10.1186/s13256-023-03765-9

**Published:** 2023-02-10

**Authors:** Alireza Rezvani, Reza Shahriarirad, Mohammad Javad Fallahi, Ali Zeighami

**Affiliations:** 1 Bone Marrow Transplantation Center, Nemazi Hospital, Shiraz University of Medical Sciences, Shiraz, Iran; 2 Thoracic and Vascular Surgery Research Center, Shiraz University of Medical Science, Shiraz, 71936-13311 Iran; 3 Student Research Committee, Shiraz University of Medical Sciences, Shiraz, Iran; 4 Department of Internal Medicine, Nemazee Hospital, Shiraz University of Medical Sciences, Shiraz, Iran

**Keywords:** Multiple myeloma, Bone marrow transplantation, Pleural effusion, Recurrence, Case report

## Abstract

**Background:**

Recurrence of multiple myeloma is among the most challenging issues for patients and treating physicians reported after autologous stem cell transplantation. However, extramedullary involvement after chemotherapy and transplantation has been rarely reported, especially as pleural manifestations. Protein electrophoresis indicated immunoglobulin A monoclonal kappa plasma cell neoplasm in our case.

**Case presentation:**

A 48-year-old middle-eastern man was referred to our clinic with cough, dyspnea, fever, and left side pleural effusion. A year after chemotherapy and autologous bone marrow transplantation, the patient presented with features in favor of pleural relapse, without bone marrow involvement. Protein electrophoresis demonstrated immunoglobulin A monoclonal kappa plasma cell neoplasm in our case. The patient was effectively treated with dexamethasone, thalidomide, cisplatin, doxorubicin, cyclophosphamide, and etoposide with no notable adverse effects.

**Conclusion:**

Physicians should be aware of various presentations of multiple myeloma relapse, especially in autologous stem cell transplantation patients. Atypical and unique presentations such as the pleural involvement warrant further reporting of evidence to provide early management and treatment options.

## Background

Extramedullary plasmacytoma is defined as monoclonal plasma cell foci outside the bone marrow that do not affect the bone marrow or have other systemic symptoms of multiple myeloma [1, 2]. With advancements in the medicine and treatment options for cancer patients, the management and treatment for recurrence of multiple myeloma (MM) are still among the most challenging issues for the patients and the treating physician. Malignant plasma cells in MM generally develop within the bone marrow, yet they can proliferate in extramedullary sites [3].

Conservative treatment for MM is still ineffective, although high-dose therapy with autologous stem cell support may be beneficial [4]. Relapses occur in 63% of autologous stem cell transplant recipients, with 4% of them being related to pulmonary diseases [5, 6]. We reported a unique instance of multiple myeloma presenting with pleural recurrence without bone marrow involvement.

## Case presentation

**Fig. 1:**
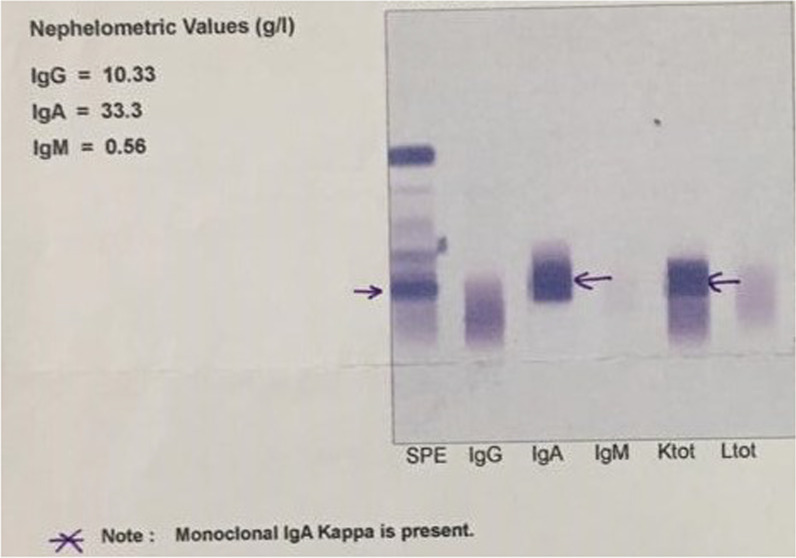
Protein electrophoresis demonstrating Immunoglobulin A (IgA) monoclonal kappa plasma cell neoplasm, arrows demonstrate the accumulation of IgA kappa

A 48-year-old Persian man was referred to our clinic with cough and dyspnea. He was relatively well until 1 year ago, with no significant past medical history, when he developed generalized bone pain, especially in the back and chest wall area. After numerous workups, a diagnosis of immunoglobulin A (IgA) monoclonal kappa plasma cell neoplasm (Fig. 1) was made, and treatment was started. Initially, the patient was treated with multiple cycles of bortezomib, dexamethasone, and lenalidomide (VRD). The patient was able to withstand all four cycles of the VRD regimen. Complete remission was obtained without any major adverse effects after the first course of therapy. After autologous stem cell transplantation, lenalidomide maintenance treatment was administered.

**Fig. 2:**
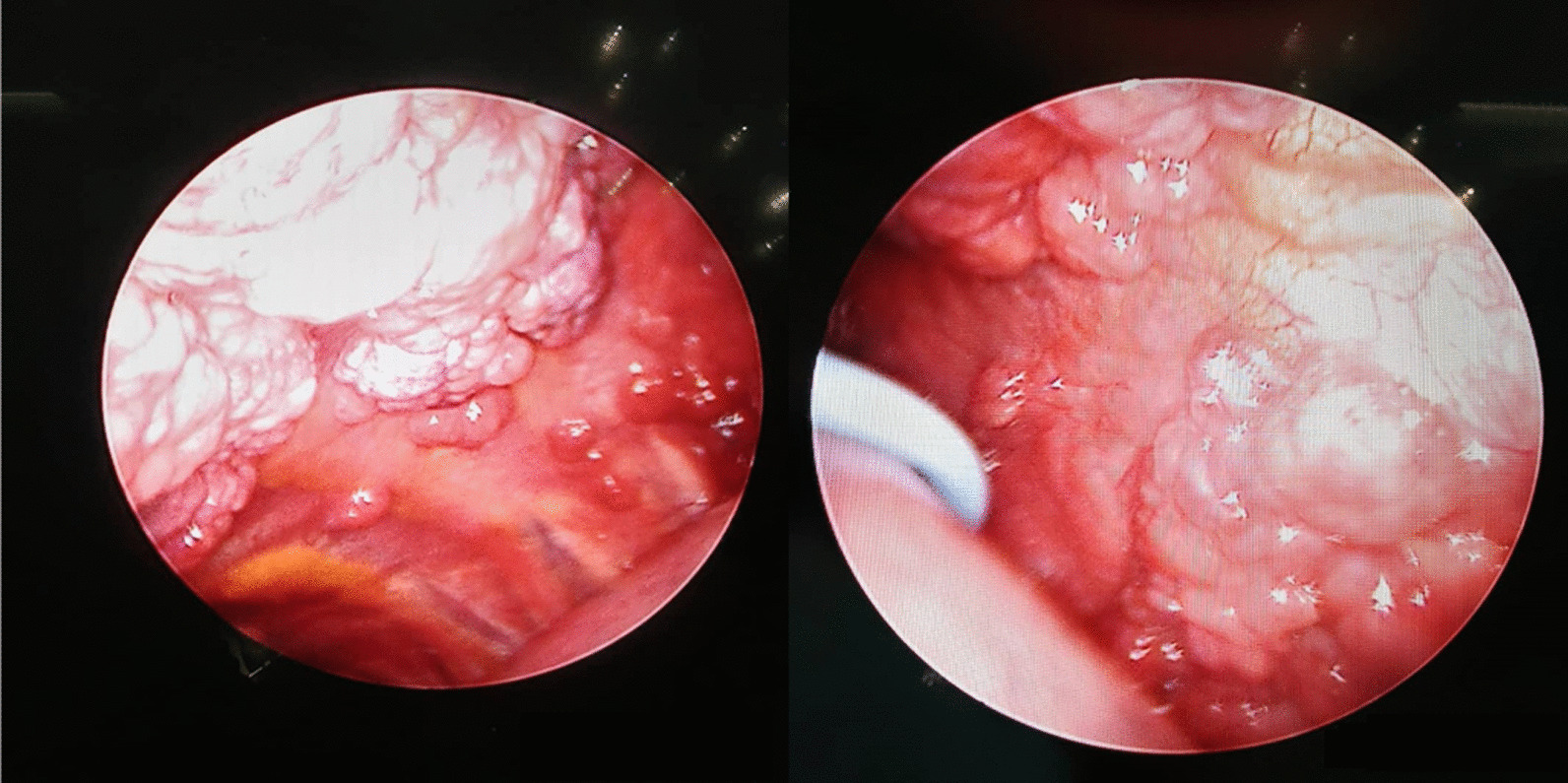
Pleuroscopy demonstrating plasmacytoma of the pleura

The patient was considered in remission until 2 months before his admission to our center, when he developed cough, dyspnea, fever, and left side pleural effusion. He underwent reevaluation with suspicion of disease relapse, in which pleural plasmacytoma was detected without bone marrow involvement. No evidence of remarkable monoclonal band was observed in the protein electrophoresis (albumin: 65.7%; gamma: 13.4%; alpha 1: 3.2%; alpha 2: 8%; beta 1: 5.5%; beta 2: 4.2%; total protein: 6.6). (Table 1) Chest sonography showed left side moderate-to-severe pleural effusion. The insertion of a pleural drainage needle catheter on the patient revealed crimson pleural fluid. Multiple polypoid nodules and masses were seen in the parietal pleura and diaphragm (Fig. 2) during pleuroscopy. A pleural biopsy of affected pleural nodules revealed plasma cell infiltration.

**Table 1:** Laboratory and paraclinical features of a male case of extramedullary relapse of Immunoglobulin A-kappa myeloma manifesting as isolated plasmacytoma of the pleura

Test	Value	Adult reference range
Immunofixation electrophoresis		
IgG	10.33	6.58–18.37
IgA	33.33	0.71–3.60
IgM	0.56	0.40–2.63
Serum protein electrophoresis		
Albumin; g/dl (%)	4.34 (65.7%)	4.02–4.76 (55.8–66.1%)
Alpha 1; g/dl (%)	0.21 (3.2%)	0.21–0.35 (2.9–4.9%)
Alpha 2; g/dl (%)	0.53 (8.0%)	0.51–0.85 (7.1–11.8%)
Beta 1; g/dl (%)	0.36 (5.5%)	0.34–0.52 (4.7–7.2%)
Beta 2; g/dl (%)	0.28 (4.2%)	0.23–0.47 (3.2–6.5%)
Gamma; g/dl	0.88 (13.4%)	0.80–1.35 (11.1–18.8%)
A/G ratio	1.92	
Total protein; g/dl	6.6	6.6–8.3
Urine biochemistry		
Urine microalbumin (random); mg/L	23	< 20
Urine creatinine (random); mg/L	151	25–400
Microalbumin/creatinine ratio	0.15	
Urine analysis		
Albumin	1+	–
Blood	1+	–
Protein	1+	–
Other		
ESR; mm/hour	18	0—15
Immunohistochemistry bone marrow		
CD138	Positive in sheet of plasma cells	
CD19	Positive in benign lymphoid nodule	
CD20	Positive in benign lymphoid nodule	
Kappa	Positive	
Lambda	Negative	

A/G: albumin to globulin; ESR: Erythrocyte sedimentation rate; IgA: Immunoglobulin A; IgG: Immunoglobulin G; IgM: Immunoglobulin M

**Fig. 3:**
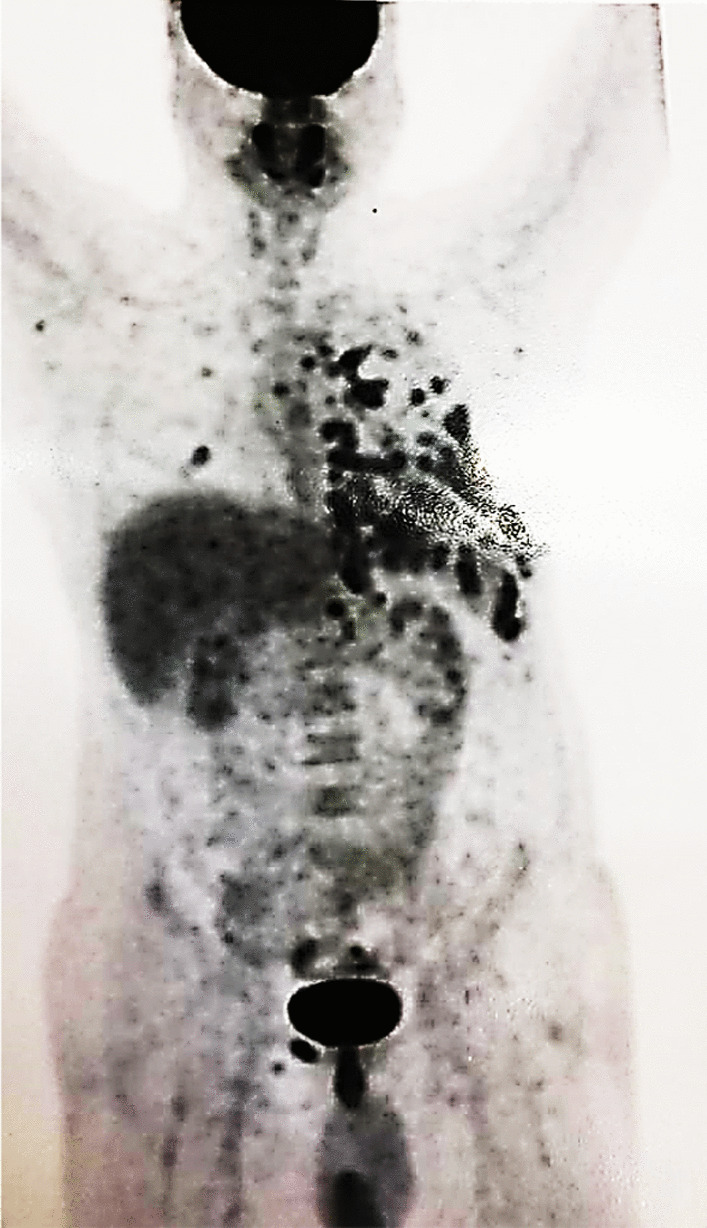
Fluorodeoxyglucose–positron emission tomography (FDG–PET) scan of a 48 year old male patient diagnosed with extramedullary relapse of IgA-kappa myeloma manifesting as isolated plasmacytoma of the pleura, demonstrating hypermetabolic multiorgan involvement in the left parietal and visceral pleura, pericardial invasion, left diaphragmatic crus lymph node involvement in thoracic (bilateral internal mammary, prevascular) and also abdominal (celiac) regions, as well as bone involvement (in T5, L4, and right pubis) and in left side oblique muscles

Fluorodeoxyglucose–positron emission tomography (FDG–PET) scan findings showed hypermetabolic multiorgan involvement in the left parietal and visceral pleura, pericardial invasion, left diaphragmatic crus lymph node involvement in thoracic (bilateral internal mammary, prevascular) and also abdominal (celiac) regions, as well as bone involvement (in T5, L4, and right pubis) and in left side oblique muscles (Fig. 3).

**Fig. 4:**
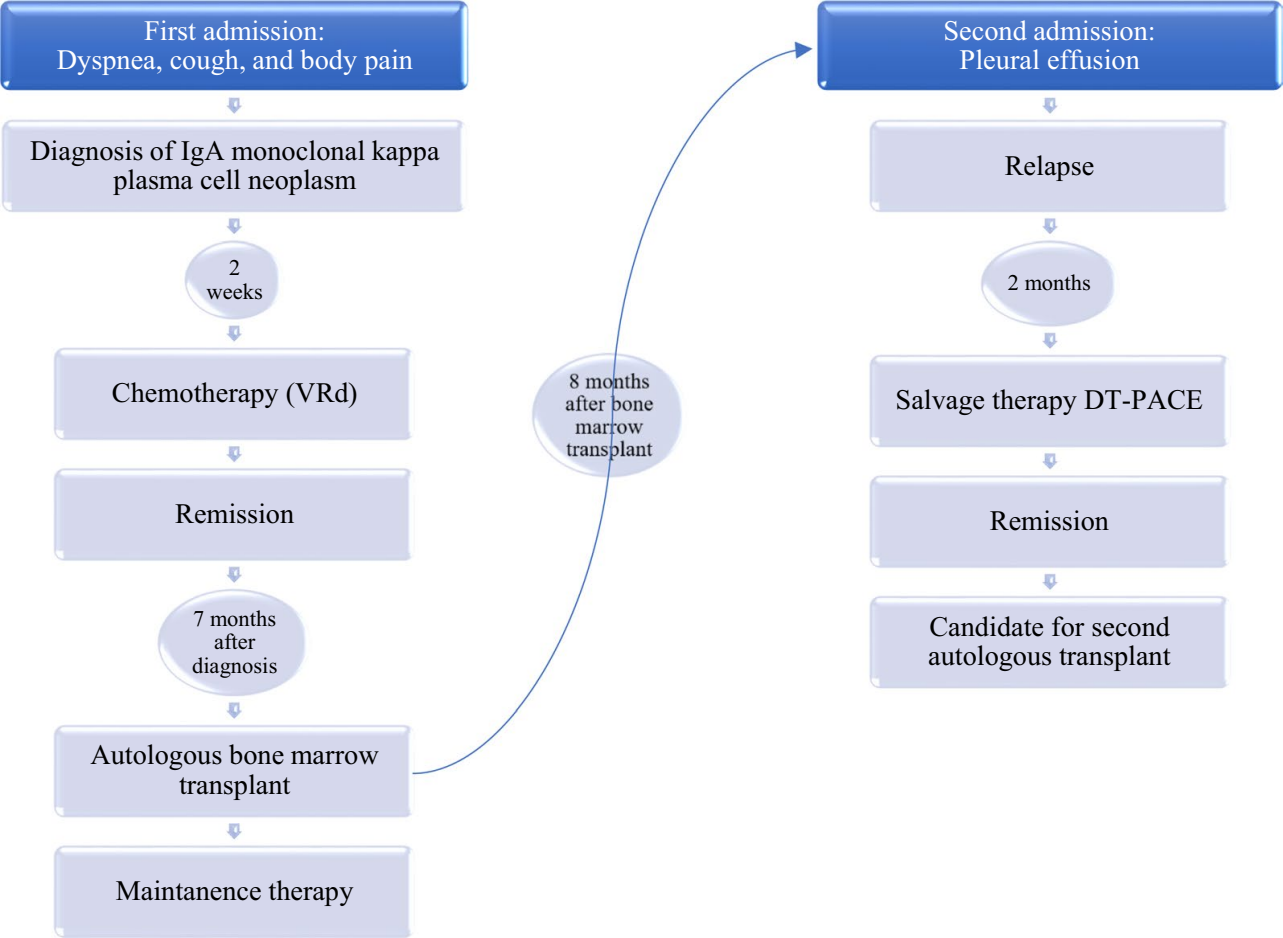
Clinical timeline of patient diagnosed with extramedullary relapse of IgA-kappa myeloma manifesting as isolated plasmacytoma of the pleura. *DT-PACE* dexamethasone, thalidomide, cisplatin, adriamycin, cyclophoshamide, and etoposide, *IgA* immunoglobulin A, *VRD* velcade (bortezomib) + revlimid (lenalidomide) + dexamethasone

The diagnosis of relapse was based on pleuroscopy and biopsy, and immunoelectrophoresis; therefore, in terms of the aggressiveness of the tumor, the patient received salvage therapy DT-PACE (a regimen consisting of six chemotherapy drugs: dexamethasone, thalidomide, cisplatin, adriamycin, cyclophosphamide, and etoposide) due to unavailability of second-line standard treatment in our center. The patient had no serious adverse effects, the medication was effective, and he became asymptomatic. After 6 months, the patient had no concerns or issues during his follow-up appointments. The patient had immunoelectrophoresis, free light chain, and beta-2 microglobulin testing as part of his follow-up examination and is now a candidate for a second autologous transplant. Figure 4 demonstrates the timeline of events in our patient.

## Discussion and conclusion

In patients with MM, autologous stem cell transplantation (ASCT) after high-dose chemotherapy increases the rate of complete response and survival compared with conventional chemotherapy. Therefore, this approach is more frequently used as the first line of therapy for eligible symptomatic MM patients under the age of 70 years, and has become the standard treatment for this group of patients. However, relapse of the disease is one of the main problems commonly reported.

Pleural plasmacytomas are extremely rare and account for around 3–6% of extramedullary disease in MM patients. Based on a recent literature review, less than ten cases of pleural plasmacytomas due to MM have so far been reported [7]. Our case reported an extramedullary relapse, particularly in the pleura, after ASCT. Other studies have reported a higher incidence of extramedullary relapse after ASCT. We believe there are several features which make our case valuable to the present literature, including the location, molecular findings, and most importantly subsequent to treatment. Our case was an extramedullary relapse in a patient who had undergone both chemotherapy and ASCT, with an initial presentation of the pleura, and respiratory symptoms of cough and dyspnea, and an immunoglobulin report demonstrating an IgA-kappa plasmacytoma. We believe that each of these three features are important and have been scarcely reported in literature. In a study by Cerny *et al*., over a 9-year period (1999–2007), only six (3.9%) out of 156 MM patients developed extramedullary relapse [8]. None of the relapses were reported in the pleura or lungs, and only two were IgA-kappa, of which one was in the liver and the other in the stomach and colon.

A report by the Spanish registry regarding 560 MM patients showed 52% relapse or progression of the disease at a median follow-up of 23 months after transplantation, with a median overall survival of 52 months and an estimated progression-free survival of 33 months [9]. One of the hypotheses for the reason for relapse is that genetic abnormalities in plasma cells induce their infiltration into areas less affected by the immune system and used for medical purposes, such as the pleura, therefore multiplying and triggering extracellular relapse. Plasmacytoma relapse without evidence of systemic myeloma progression, occurring either as an isolated phenomenon or concurrent with systemic relapse, has been reported in ASCT and allogeneic bone marrow transplantation (BMT), ranging from 7% to 35% [10–12]. The most frequent (66%) pattern of relapse is the classical pattern, presenting as a progressive increase of monoclonal component and medullary plasmacytic infiltration.

Pleural involvement is rarely mentioned in the medical literature, especially after treatment. Zeiser *et al*. reported extramedullary recurrence in the lungs, soft tissue, bone, and pericardium after ASCT, and also in the lungs, soft tissue, skin, central nervous system, and pericardium following allogeneic BMT [5]. Feng *et al*. [13] reported solitary pleural plasmacytomas, which presented with massive pleural effusion without evidence of monoclonal gammopathy after local radiotherapy and systemic chemotherapy. Saidane *et al*. reported a case of pleural involvement after treatment of cutaneous MM [14]. Also, Harith Al-Ataby *et al*. [15] reported a case of pleural plasmacytomas due to MM relapse that was asymptomatic. However, their case was 9 months after chemotherapy and radiotherapy, while in our case, the relapse was detected 8 months after ASCT and VRD therapy.

In the Spanish registry study, extramedullary recurrence was identified in around 7% of patients who received ASCT (14% of relapsed patients), with numerous or single lesions presenting with null or minimal paraprotein. [9] Terpos *et al*. also reported that 9.5% of patients who underwent ASCT and 6% of those who received an allograft BMT relapsed as an extramedullary plasmacytoma. [10] Allogeneic BMT is linked with an immunologic graft-versus-myeloma effect, therefore, it is expected to be accompanied by lower rates of disease recurrence. [16–18]

Protein electrophoresis demonstrated IgA monoclonal kappa plasma cell neoplasm in our case. Previous reports of extramedullary relapse of IgA-lambda myeloma were reported as intracranial plasmacytomas or pancreatic involvement [19, 20]. A report by Boyle on the impact of severe isotype paired suppression, as measured by IgA Hevylite, on survival, showed that severe hypogammaglobulinemia indicates a dismal prognosis in patients with IgA MM, and that IgA isotype paired suppression is prognostic at the time of presentation among patients with IgA MM [21]. Furthermore, Alyea *et al*. reported no significant correlation between IgA isotype with progression-free survival and overall survival [4].

The treatment of extramedullary plasmacytomas in the setting of MM relapse is rather challenging. Regarding the aggressiveness of the tumor, the patient received salvage therapy DT-PACE (a regime consisting of six chemotherapy drugs: dexamethasone, thalidomide, cisplatin, adriamycin, cyclophosphamide, and etoposide), due to unavailability of second-line standard treatment such as carfilzomib in our center. The patient developed no significant side effects, and the treatment was successful. The DT-PACE treatment is an effective combination chemotherapy for high-risk patients like ours. In 2003, Lee *et al*. indicated that this regimen may induce a 32% partial response and a 16% full and near-complete response in high-risk and advancing patients with cancer [22]. Carfilzomib is a second-generation proteasome inhibitor. The phase 3 ENDEAVOR study included a combination of carfilzomib and dexamethasone (KD) [23]. The carfilzomib-lenalidomide-dexamethasone (KRD) and lenalidomide-dexamethasone therapies were evaluated in the ASPIRE study [24]. The results of both studies supported using carfilzomib as a means of extending life. Our treatment followed guidelines reported by the National Comprehensive Cancer Network (NCCN) and Oxford Myeloma group [25, 26]. As mentioned in the NCCN, we initially treated our patients with VRD therapy. During the recurrence, despite the fact that current guidelines recommended triplet regimens (NCCN) for refractory patients, we chose the DT-PACE combination for our patient due to the lack of second-line treatment options in our center, and also the aggressiveness of the tumor based on the patients dyspnea as well as the course of the disease [25, 26]. Carfilzomib is a highly successful treatment for extramedullary plasmacytomas such as MM. A KD chemotherapy treatment was found to be beneficial for a patient with pleural and cardiac involvement by Espaol *et al*. in 2017 [27]. In a separate case report, the authors noted that the combination of carfilzomib and lenalidomide was effective in plasmacytomas when administered without radiation to a patient with brain involvement [28]. Three months of DT-PACE therapy resulted in a complete response in our patient. Despite introducing several new treatments, MM management is still a challenging issue.

In conclusion, this case is remarkable since our patient received both chemotherapy and ASCT due to IgA-kappa MM, but still relapsed with extramedullary manifestations, particularly pleural involvement, which is a rare type and was remarkably observed in the FDG–PET scan. The prevalence of extramedullary MM relapses has grown in recent years, and clinicians should be familiar with the many manifestations of this illness, particularly in ASCT patients, to give early management and treatment options.

## Data Availability

All data regarding this case has been reported in the manuscript. Please contact the corresponding author if you are interested in any further information.

## Abbreviations

ASCT: Autologous stem cell transplantation
BMT: Bone marrow transplantation
DT-PACE: Dexamethasone, thalidomide, cisplatin, doxorubicin, cyclophosphamide, etoposide
FDP–PET: Fluorodeoxyglucose–positron emission tomography
IgA: Immunoglobulin A
MM: Multiple myeloma
